# Stress distribution and bone deformation around narrow-diameter implants at different placement depths and in different bone densities

**DOI:** 10.1590/1807-3107bor-2026.vol40.050

**Published:** 2026-07-20

**Authors:** Fabricia Teixeira BARBOSA, Vanessa Felipe VARGAS-MORENO, Raissa Micaella MARCELLO-MACHADO, Suzana Peres PIMENTEL, Mônica Grazieli CORRÊA, Mabelle Freitas MONTEIRO, Fabiano Ribeiro CIRANO, Ricardo Armini CALDAS, Márcio Zaffalon CASATI

**Affiliations:** (a) Universidade Paulista – Unip, School of Dentistry, Dental Research Division, Sao Paulo, SP, Brazil.; (b) Universidade Federal de Santa Catarina – UFSC, Department of Dentistry, Florianópolis, SC, Brazil.

**Keywords:** Dental Implants, Finite Element Analysis, Bone Density, Dental Stress Analysis

## Abstract

This study evaluated the biomechanical behavior of narrow-diameter Morse taper implants (3.5 mm) placed in bone of different densities and at different depths, using finite element analysis. Sixteen three-dimensional virtual models, meshed with tetrahedral elements, were created. Each model comprised cortical bone, cancellous bone, a Morse taper implant, an abutment, and a single maxillary central incisor crown. An oblique force (30 degrees buccally) of 150 N was applied to evaluate peri-implant stress distribution across four bone densities (D1, D2, D3, and D4) and at four placement depths (0, 1, 2, and 3 mm). The interfaces were modeled as perfectly bonded, except at the implant-abutment interface (friction coefficient = 0.3). All materials were assumed to be linearly elastic and isotropic. After a nonlinear analysis, the maximum and minimum principal stresses, maximum principal strain, and Mohr-Coulomb ratio were recorded. The results revealed a stress distribution pattern with lower peak stress values at shallower placement depths and in D1 bone. All groups showed a similar trend: as the implant placement depth increased, the point of greatest stress concentration shifted apically. Implant placement in cortical bone provided a protective effect under oblique loads. In D3 and D4 bone types, crestal placement showed better biomechanical performance than subcrestal placement.

## Introduction

Bone characteristics at an edentulous site play a critical role in implant-supported rehabilitation, particularly in treatment planning, which includes selecting the implant, determining the surgical approach, predicting primary stability, estimating healing time, and planning provisional loading during prosthetic reconstruction.^1-3^ Because bone-to-implant contact is greater in cortical bone than in cancellous bone,^4^ achieving successful osseointegration requires evaluating not only bone volume but also bone density.^5^ This assessment is critical in regions with high cortical heterogeneity, such as the maxillary central incisor area, where insertion torque may exceed the implant’s structural tolerance, suggesting that cortical engagement depth may influence the biomechanical risk threshold of narrow-diameter implants.

Higher bone density is associated with improved primary implant stability and more favorable stress dissipation at the bone-to-implant contact interface during function.^6^ However, overload may trigger peri-implant bone resorption, peri-implantitis, and possible implant failure or loss.^6^ Lekholm and Zarb’s 1985 classification^6^ describes four bone densities: Type I (D1), highly cortical and typical of the anterior mandible; Type II (D2), cortical bone surrounding a cancellous core, common in the posterior mandible; Type III (D3), thinner cortical plates encasing cancellous bone, often found in the anterior maxilla; and Type IV (D4), low-density cancellous bone, typically observed in the posterior maxilla. Additionally, when planning anterior implants, soft-tissue thickness influences subcrestal placement decisions and the mechanical environment preceding cortical load transfer, emphasizing the need to evaluate bone and soft-tissue parameters together for optimal positioning in the esthetic zone.

The rate of osseointegration is strongly affected by bone density and trabecular organization. Implants placed in dense cortical bone commonly achieve secondary stability within 6–8 weeks, whereas implants placed in low-density trabecular bone may require 12–16 weeks to achieve comparable fixation.^7^ Dense cortical substrates also have biological limitations, including reduced medullary vascularization, which may compromise early nutrient supply to the clot and impair woven bone formation.^8^ High mechanical resistance at these sites often leads to high insertion torque, which improves primary stability but increases susceptibility to cortical microdamage and transient ischemia, thereby compromising early peri-implant bone homeostasis.^3,9^ Wolff’s Law supports this mechanobiological adaptation, whereby bone develops the structure most suited to resist the forces acting upon it, reinforcing the idea that implant positioning may modulate long-term peri-implant bone remodeling.^10^

Implant failures in low-density bone are frequently attributed to incomplete osseointegration, suboptimal three-dimensional implant positioning,^11^ occlusal cantilevers,^7^ overload or parafunctional habits,^6^ and soft-tissue complications.^6,12^ Implant-abutment connection design may also affect stability, osseointegration, and stress distribution.^13,14^ Morse taper implants allow bone formation over the platform, which enhances mechanical performance, promotes a more predictable biological seal, improves esthetic outcomes, and supports long-term marginal bone preservation^15^, directly contributing to peri-implant tissue stability and crestal bone preservation.^15^

Despite clinical recommendations for subcrestal placement of Morse taper implants, local anatomy may limit platform depth, and there is no consensus on the ideal vertical position.^16-19^ Peri-implant soft-tissue thickness helps determine subcrestal placement depth,^4^ as it modulates the mechanical environment before cortical load transfer, emphasizing the need to consider osseous and soft-tissue parameters jointly for optimal positioning in the esthetic zone.^3,11,17^ Variations in cortical thickness and cancellous density may compromise stress distribution, increasing the risk of biomechanical failure.^14,15^ Previous studies have shown that the maxillary central incisor region often has heterogeneous bone, commonly with trabecular-dominant substrates and thin cortical plates, especially in cases of crestal or buccal resorption.^3,7,11^ These profiles exhibit lower trabecular modulus and greater cortical strain under oblique loading,^20^ which affects biomechanical behavior as platform depth increases. Thus, this study used finite element analysis (FEA) to evaluate the biomechanical behavior of narrow-diameter implants (3.5 mm) placed at different depths and in bone of different densities to inform the development of a biomechanical protocol for clinical practice. The null hypothesis was that neither placement depth nor bone density would affect the biomechanical behavior of narrow-diameter implants.

## Methods

**Figure 1:**
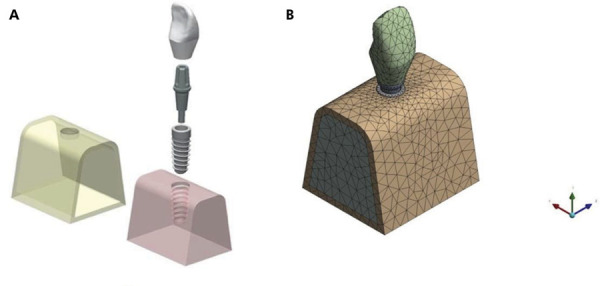
(A) Exploded view of virtual model: cortical and cancellous bone; implant; abutment; crown. (B) Mesh model.

FEA was used to simulate the restoration of a maxillary central incisor with a narrow-diameter Morse taper implant, featuring a prosthetic platform 1.5 mm above the bone crest. Virtual models were created in SolidWorks (Dassault Systèmes, SolidWorks Corp.), with the following components: cortical bone, cancellous bone, a Morse taper implant, an abutment, and a single maxillary central incisor crown (Figure 1A).

**Figure 2:**
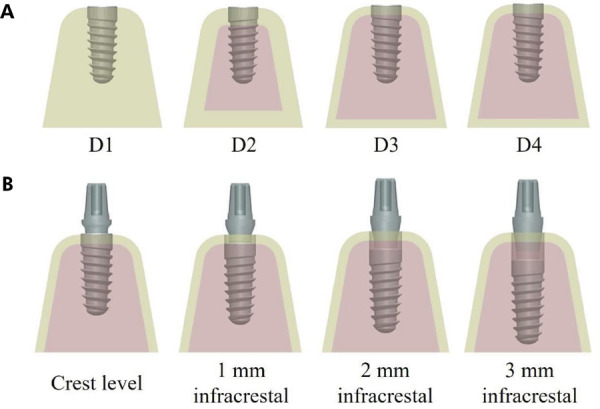
(A) Simulated bone: D1; D2; D3; D4. (B) Implant placement depth in type D3 bone. Gray – cortical bone; Pink – cancellous bone.

The geometry of the bone components varied according to bone density: Type I bone (D1), composed entirely of cortical bone; Type II bone (D2), with a 2-mm-thick cortical layer; and types III (D3) and IV (D4), with a 1-mm-thick cortical layer^4^ (Figure 2A). Bone dimensions were standardized to 14 mm in height, 16 mm in length, 14 mm in width at the base, and 5.5 mm at the crest. An implant (3.5 × 9 mm, DueCone; Implacil de Bortoli) and an F138 stainless steel abutment^21^ (3.3 × 4 × 1.5 – 4.5 mm, Ideale; Implacil de Bortoli) were modeled according to the manufacturer’s specifications. The abutment transmucosal height, ranging from 1.5 mm to 4.5 mm depending on the placement depth (crest level, 1 mm, 2 mm, and 3 mm), was selected to standardize the position of the prosthetic platform at 1.5 mm above the alveolar bone crest (Figure 2B).

The models were assembled into 16 groups by varying placement depth and bone density: D1.0 (D1 bone, 0 mm depth); D1.1 (D1 bone, 1 mm depth); D1.2 (D1 bone, 2 mm depth); D1.3 (D1 bone, 3 mm depth); D2.0 (D2 bone, 0 mm depth); D2.1 (D2 bone, 1 mm depth); D2.2 (D2 bone, 2 mm depth); D2.3 (D2 bone, 3 mm depth); D3.0 (D3 bone, 0 mm depth); D3.1 (D3 bone, 1 mm depth); D3.2 (D3 bone, 2 mm depth); D3.3 (D3 bone, 3 mm depth); D4.0 (D4 bone, 0 mm depth); D4.1 (D4 bone, 1 mm depth); D4.2 (D4 bone, 2 mm depth); and D4.3 (D4 bone, 3 mm depth). These models were imported into the FEA software (Ansys Workbench; ANSYS Inc.), where material properties were assigned to each component (Table). All materials were assumed to be isotropic, homogeneous, and linearly elastic.^17^ A convergence analysis (5% tolerance) was performed to ensure adequate precision of the results. Meshes with 202,000 to 213,000 nodes and 137,000 to 145,000 10-node tetrahedral (TET10) elements were generated and evaluated for element quality (Figure 1B). Contact between all components was modeled as bonded,^18,22^ except at the implant-abutment interface, where frictional contact was applied (µ = 0.3). For fixed supports (zero degree of freedom), the thinned areas (mesial and distal) of the bone were modeled as fixed supports.^19^ In all models, a 150-N static load was applied at a 30-degree angle to the long axis of the implant in the buccal direction.

**Table:** Mechanical properties of the materials.

Material	Elastic modulus (GPa)	Poisson’s ratio
Feldspathic ceramic^13,26^	82.8	0.35
Metal framework^26^	218.0	0.33
Resin cement^26^	7.0	0.25
Abutment^14^	187.5	0.33
Implant^13^	110.0	0.35
Dense cancellous bone (Types II and III)^10^	1.37	0.30
Low-density cancellous bone (Type IV bone)^10^	1.10	0.30
Cortical bone^12^	13.7	0.30

The analysis was nonlinear due to frictional contact. All simulations were assessed quantitatively and qualitatively. The results were evaluated separately for the structures of primary interest: cortical and cancellous bone. Values for maximum principal stress (σ_max_), minimum principal stress (σ_min_), maximum principal strain, and Mohr-Coulomb stress ratio (σ_MC ratio_) were recorded.^15,22, 23^ In the quantitative analysis, peak values were recorded for each parameter. In the qualitative analysis, color gradients were assigned based on the values at each data point.^24^Physiological reference limits for maximum and minimum principal stresses were set at 54.8 MPa for cortical bone and 5.48 MPa for cancellous bone.^20,25^

## Results

**Figure 3:**
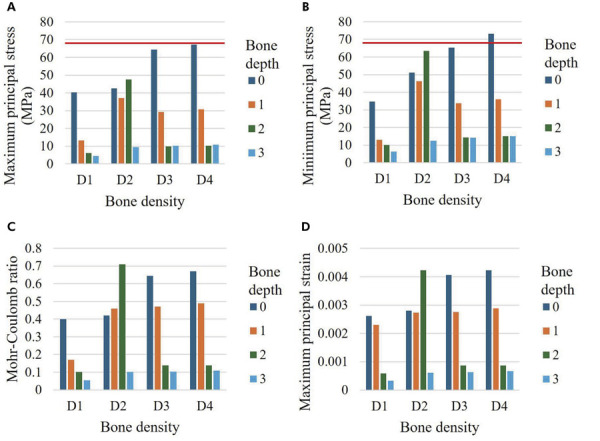
Cortical bone: (A) Maximum principal stress; (B) Minimum principal stress; (C) Mohr-Coulomb ratio; (D) Maximum principal strain versus bone density. Red line: physiological reference limits for maximum and minimum principal stresses.

**Figure 4:**
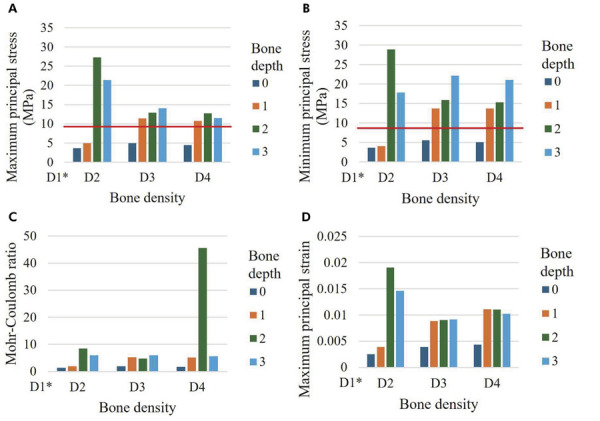
Cancellous bone: (A) Maximum principal stress; (B) Minimum principal stress; (C) Mohr-Coulomb ratio; (D) Maximum principal strain versus bone density. Red line: physiological reference limits for maximum and minimum principal stresses. *Cancellous measurements were not analyzed in D1 because D1 consists entirely of cortical bone.

The quantitative results for the different bone densities (D1–D4) and depths (0, 1, 2, and 3 mm) are shown in Figures 3 and 4. Figure 3 demonstrates distinct cortical behavior across four bone densities. In D1, even at the crest level, σ_max_ remained below the physiological limit. In contrast, crest-level placement in D2, and especially in D3 and D4, resulted in values approaching or exceeding the cortical limit. Increasing the subcrestal placement depth (1–3 mm) consistently reduced this stress in D2–D4, with a stronger protective effect at 2–3 mm. σ_min_ followed the same pattern and increased in D3 and D4 at the crest, exceeding physiological limits. The σ_MC ratio_ reinforced this trend; in D1 and D2, values remained low at all depths, whereas in D3 and D4, crestal placement resulted in higher values. Subcrestal placement at 2–3 mm substantially reduced this ratio, with progressive improvement as depth increased.

As shown in Figure 4, σ_max_ in cancellous bone approached or exceeded physiological limits mainly in D2 and D3 at the crestal level. A 2-mm depth produced the greatest stress reduction in cancellous bone, especially in D3 and D4. σ_min_ decreased with subcrestal placement. The σ_MC ratio_ of cancellous bone was generally lower than that of cortical bone. However, in D3 and D4, crestal placement produced high peaks, occasionally exceeding 40 in D4. Subcrestal positioning reduced these values, particularly at 2 mm.

**Figure 5:**
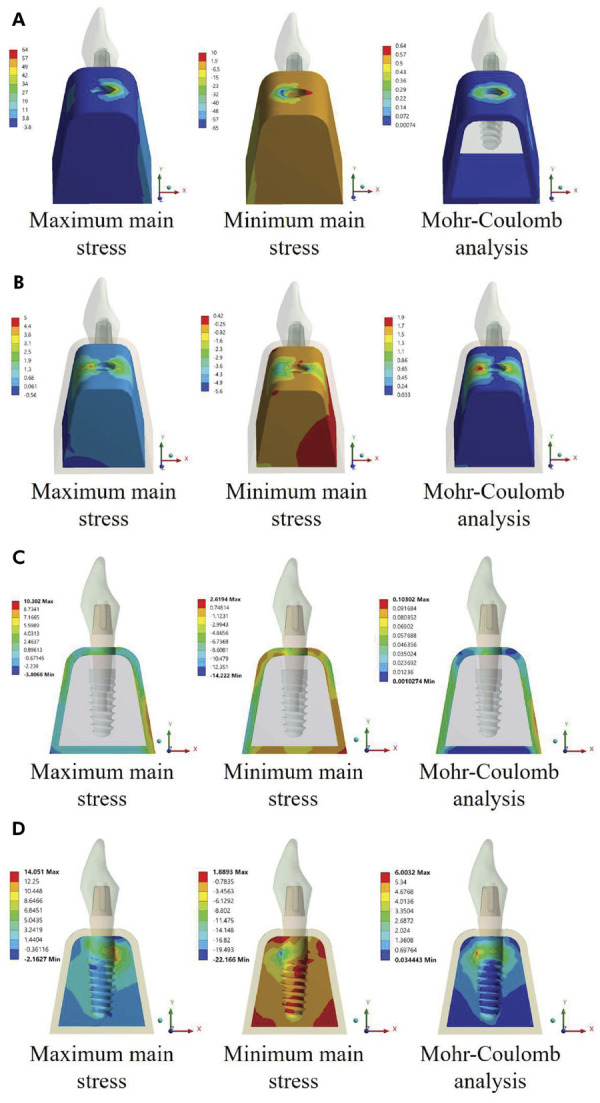
Qualitative analysis in D3.0: Maximum principal stress; Minimum principal stress; Mohr-Coulomb analysis for cortical (A) and cancellous bone (B). Qualitative analysis in D3.3: Maximum principal stress; Minimum principal stress; Mohr-Coulomb analysis for cortical (C) and cancellous bone (D).

Qualitative results for groups D3.0 and D3.3 are shown in Figure 5. In D3.0, higher σ_max_ values were observed around the buccal bone crest, indicating load concentration near the implant interface. σ_min_ also revealed a broader cervical compression zone. The σ_MC ratio_ showed extensive high-risk areas, reinforcing the quantitative findings. In D3.3, the maps showed reductions in σ_min_ and σ_max_ in cortical bone, with regions of peak stress shifting apically. The σ_MC ratio_ indicated a lower risk of cortical and cancellous failure, with substantially smaller critical areas compared with D3.0.

## Discussion

Implant characteristics and properties have been extensively studied to enhance rehabilitation outcomes, and factors such as bone density have been shown to be critically important for success.^2^This study analyzed the effects of varying implant placement depths in bone of different densities, given the ongoing debate in the literature regarding stress distribution around the implant neck. Moreover, stress concentrations above the yield strength may cause cortical microfractures and induce bone resorption.^3^ The results of this study demonstrate that structural bone characteristics, particularly density, significantly influence stress distribution; therefore, the null hypothesis was rejected. Previous studies have demonstrated that regions with different densities show distinct stress concentration patterns, with lower-density bone showing higher stress accumulation.^2,14^ The findings of this study reinforce previous in silico analyses showing that crestal cortical bone is consistently subjected to the highest stresses, especially under oblique loading.^26^

These biomechanical patterns reinforce the idea that cortical engagement and implant body diameter play decisive roles in determining the structural stress threshold tolerated by the fixture, which is clinically relevant when narrow-diameter implants are subjected to high loads. ^27, 28^ Therefore, this study provides new biomechanical insight by presenting a structural rationale for diameter-dependent torque-induced fracture susceptibility, demonstrating how cortical resistance can critically reduce the mechanical safety margin of narrow-diameter implants placed at clinically relevant subcrestal depths. Additionally, cortical engagement must be interpreted alongside peri-implant soft-tissue thickness, as mucosal dimensions influence the clinical determination of subcrestal positioning, selection of transmucosal components, and modulation of initial load dissipation before force transmission into the cortical interface.^4,16,17,19^

Narrow-diameter implants show mechanical resilience limitations, which are clinically reflected in higher biological and structural complication rates, particularly in dense cortical substrates that generate excessive insertion torque, matching this study’s stress distribution.^26,27^ Evidence indicates higher mechanical failure rates for narrow-diameter implants than for conventional ones, with complication rates ranging from 2.2%–17.28%, depending on the loading protocol and follow-up period.^29,30^ Their reduced metal cross-section increases fatigue fracture susceptibility and cervical stress concentration under oblique loading, which is consistent with finite element failure modes.^27^ Dense, highly corticalized bone, often produces excessive insertion torque in Type I like substrates, generating high frictional resistance, cumulative fatigue stress, and reduced safety margins, especially in narrow implant bodies.^3,9^ *In vitro* studies have shown compromised endurance when metallic systems face excessive locking resistance or notch-related stress risers, supporting clinical reports of implant fracture after high torque demand in dense cortical bone, particularly for narrow-diameter implants.^31,32^ Although high torque may improve primary stability, exceeding the alloy’s fatigue tolerance markedly increases the risk of structural failure, requiring a balance between achieving cortical engagement and reducing safety margins in narrow-diameter implants.^18,19^

These clinical fracture and survival trends support the mechanobiological rationale of our findings, suggesting that the stress distribution patterns and torque-related risks predicted by our model also provide a structural explanation that may guide implant selection across implant designs and placement depths. In D1, all cortical parameters decreased as depth increased, indicating that subcrestal positioning was more favorable for D1. It is important to note that cancellous measurements were not analyzed in D1 because it consists entirely of cortical bone, and cortical characteristics were applied to this region. These findings are consistent with previous studies showing that a thicker cortical layer promotes improved stress distribution around implants.^2,9,19^ Nonetheless, dense cortical bone poses biological and mechanical concerns, including excessive cortical locking, reduced microvascular supply, and limited local perfusion, which may delay remodeling and compromise healing.^1,3^ High interfacial friction in highly cortical substrates may also result in elevated insertion and removal torque, increasing the risk of cortical microdamage and ischemic compression.^3,9^ When narrow-diameter implants are used, these risks become more critical, as their smaller metal bodies offer lower structural resistance, heightening fracture susceptibility under excessive torque or concentrated cortical loading.^27^ Therefore, although cortical engagement enhances stress shielding, implant selection in dense cortical bone must account for tissue hypovascularity and mechanical vulnerability of narrow-diameter implants.^8^

Building on this mechanobiological interpretation, implant macrogeometry is another relevant factor, as different designs may respond differently to cortical engagement and stress patterns. In D2, all cortical parameters decreased with increasing depth, except at 2 mm, where stress increased because the implant was positioned immediately below the cortical layer, thereby reducing stability. In cancellous bone, these values increased at all depths except 3 mm. At depths of 2 mm and greater, stress values exceeded physiological thresholds (54.8 MPa for cortical bone and 5.48 MPa for cancellous bone),^9,11,20^ highlighting the importance of placing the implant within the cortical bone, which is stiffer and has a higher modulus of elasticity.^2,14^ In D3 and D4, all cortical parameters decreased as depth increased, whereas cancellous bone parameters progressively increased at deeper positions. These results indicate that increasing depth shifts stress away from the cortical bone toward the cancellous bone, with potential implications for stability and adaptation depending on bone density. D3 and D4 behave similarly because they share the same cortical thickness, the only difference being the lower elastic modulus of D4 cancellous bone. In D3.0, values were 10% above the critical threshold for cortical bone,^20,26^ with a small peak stress area in the cervical third. In D3.3, σ_max_ and σ_min_ values were 280% and 400% above the threshold for cancellous bone,^20,25^ respectively, with larger peak stress areas. Thus, crest-level placement showed superior performance, consistent with previous studies.^2,18^

The σ_MC ratio_ is a reliable parameter for evaluating bone structure—a brittle material—as it relates traction and compressive stresses at a given point.^22,23^ A ratio above 1 indicates a considerable risk of bone damage, potentially leading to remodeling and local microfractures.^3,15^ The σ_MC ratio_ in cancellous bone exceeded 1 for all groups at all depths but was below 1 in cortical bone. In D3.0, only a small region had a ratio that slightly exceeded 1, whereas D3.3 showed a well-defined region with a ratio greater than 1. This illustrates the protective effect provided by cortical bone when the implant engages the cortical layer,^2,14,15^attributable to its stiffness and higher elastic modulus.

This protective effect is an important consideration in implant-supported rehabilitation in the esthetic zone, such as the clinical scenario simulated in this study. Buccal bone is highly susceptible to resorption, which is influenced by load intensity and direction, residual bone thickness, and density, with greater thickness and density yielding better outcomes.^9^ Chu et al.^19^ supported these findings, reporting that increased cortical thickness reduced stress and strain by 22%–49% at both crestal and subcrestal positions. A linear decrease in stress with increasing depth was also observed, with lower stress in thicker cortical bone, consistent with previous analyses,^2,11^ even in low-density cancellous bone.^33^A previous study highlighted the importance of adequate bone density around the implant neck to prevent microfractures, as this region experiences the highest stress, with much greater Von Mises stress concentrations in low-density bone.^5^ Poovarodom et al,^17^ also supported our findings, showing how implant depth influences bone remodeling through density changes. Conversely, Santiago et al.^15^ reported no significant differences between D3 and D4 in various implant designs under oblique loading; however, this may be attributed to the uniform cortical thickness used in their models, as D1 and D2 were excluded.

Previous studies have also evaluated three-dimensional implant positioning. Placement depth may vary for several reasons, including esthetic considerations. Previous studies have shown favorable outcomes for crest-level placement,^13,18^ as evidenced by higher Von Mises stress in subcrestal positions.^18^ Conversely, other studies contradict these findings, reporting a more favorable stress distribution for subcrestal placement.^16,34^ Sotto-Maior et al.^16^ reported favorable subcrestal results when evaluating σ_min_ and strain in cortical and cancellous bone with crestal and 2-mm-subcrestal implants, with or without bicortical fixation. In contrast, Chou et al.^34^ showed that the depth of implant placement had no significant effect on bone stress.

Hence, there is no consensus regarding the interaction between bone density and placement depth, making interpretation and comparison challenging. Furthermore, each model have its own mesh, boundary conditions, contact, and analysis parameters, which generate unique results. Therefore, this study considered different placement depths and bone densities, which enabled us to evaluate a scenario and not just a three-dimensional representative model of an isolated condition since preventing peri-implant bone resorption is crucial in the long term. As bone remodeling is mechanically driven, and despite its complexity, FEA remains a powerful method for studying complex situations while isolating variables, showing 95% reliability,^28^a level of accuracy not achievable clinically or *in vitro*. Further studies are needed to support the ongoing development and refinement of implant materials and to better understand their long-term behavior, especially in D4 bone.

## Conclusion

Implant placement in cortical bone provided a protective effect under oblique loads. In D3 and D4 bone, crestal placement showed better biomechanical performance than subcrestal placement.

## Data Availability

The authors declare that all data generated or analyzed during this study are included in this published article.
